# 419. Viral SARS-CoV-2 rebound rates in linked commercial pharmacy-based SARS-CoV-2 RT-qPCR testing and healthcare claims

**DOI:** 10.1093/ofid/ofad500.489

**Published:** 2023-11-27

**Authors:** Shishi Luo, Lisa M McEwen, Scott P Kelly, Magnus Isaksson, Sarah Murphy, Simon White, J T McCrone, Geraint Levan, Sharad Santhanam, Mary Lynn Baniecki, Candace Bramson, Heather Rubino, Victoria Hendrick, Holly Soares, Jennifer Hammond

**Affiliations:** Helix, San Mateo, California; Helix, San Mateo, California; Pfizer, New York, New York; Helix, San Mateo, California; Formerly Helix, Boston, Massachusetts; Helix, San Mateo, California; Helix, San Mateo, California; Helix, San Mateo, California; Helix, San Mateo, California; Pfizer Inc, Cambridge, Massachusetts; Pfizer Inc, Cambridge, Massachusetts; Pfizer, New York, New York; Pfizer, New York, New York; Pfizer, New York, New York; Pfizer Inc, Cambridge, Massachusetts

## Abstract

**Background:**

Viral SARS-CoV-2 RNA rebound has been described as a positive viral antigen or PCR test that follows a negative test. Although early descriptions of this phenomenon were linked to nirmatrelvir/ritonavir (NMV-r) treatment, subsequent studies have shown that viral rebound occurs both in untreated patients and patients treated with other antiviral therapies and is not associated with progression to severe disease. The rate of viral SARS-CoV-2 rebound has not been well characterized in large cohorts outside of clinical trials.

Schematic representation of study population

**Figure:**
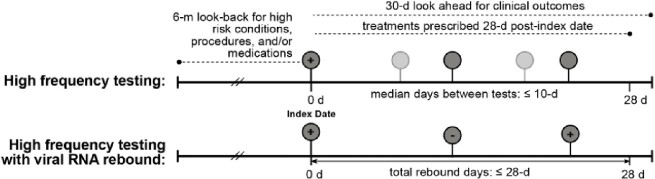

(Upper) High frequency testing and (Lower) High frequency testing with viral RNA rebound, along with relevant clinical information indicated by dashed lines. Each circle represents a SARS-CoV-2 RT-qPCR test with the darker shade indicating the minimum required tests to meet the definition and the lighter shade to reflect the possibility of >3 tests. The index date is the first positive test within 28-days.

**Methods:**

In this large retrospective US-based cohort study, RT-qPCR SARS-CoV-2 test data were linked to healthcare claims to calculate the viral rebound rate in treated and untreated individuals, among subjects tested at least 3 times during a SARS-CoV-2 infection. This cohort of 30,646 unique patients includes SARS-CoV-2 vaccinated patients, patients infected during the Omicron era, and high risk patients (those with underlying medical conditions associated with a higher risk for severe SARS-CoV-2 infection).

**Results:**

The observed rate of viral SARS-CoV-2 RNA rebound was approximately 3.5% in NMV-r treated infections compared to 1.5% in untreated infections during the Omicron era. Viral rebound in vaccinated, high-risk, or older patients occurred at comparable rates to the overall cohort. Viral rebounds to high RNA levels in NMV-r treated infections were rare (8% of viral rebounds) and comparable in frequency to those in untreated infections (11% of viral rebounds) during the Omicron era.

Viral RNA rebound rates

**Figure:**
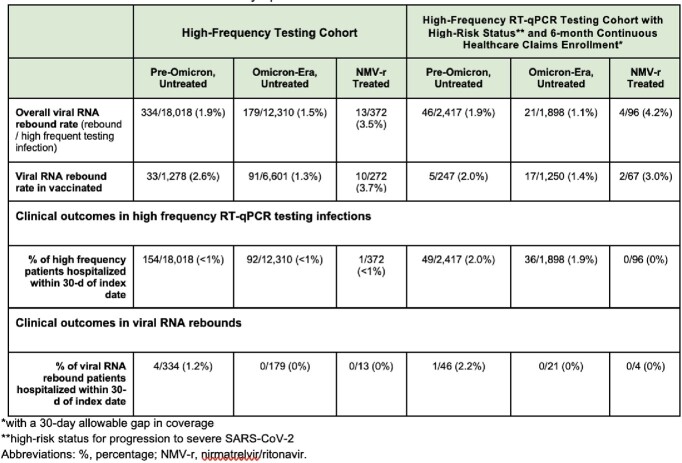

Viral RNA rebound rates and characteristics of the patient cohort, stratified by risk group, Omicron era, and NMV-r treatment. Untreated groups exclude patients who received NMV-r or a different SARS-CoV-2 treatment 28 days post-index date.

**Conclusion:**

This study demonstrates that SARS-CoV-2 viral RNA rebound is rare and occurs in both treated and untreated SARS-CoV-2 infections, with rates comparable to those seen in the EPIC-HR clinical trial for NMV-r. Even when viral rebound occurs, only a small minority of cases rebound to a high viral RNA level and rarely result in progression to severe disease.

**Disclosures:**

**Shishi Luo, PhD**, Helix: Employee **Lisa M. McEwen, PhD**, Helix: Employee **Scott P. Kelly, PhD**, Pfizer: Employee|Pfizer: Stocks/Bonds **Magnus Isaksson, PhD**, Helix: Employee **Sarah Murphy, n/a**, Helix: Former employee **Simon White, n/a**, Helix: Employee **JT McCrone, PhD**, Helix: Employment **Geraint Levan, n/a**, Helix: Employee **Mary Lynn Baniecki, PhD**, Pfizer Inc: Employee|Pfizer Inc: Stocks/Bonds **Candace Bramson, MD**, Pfizer Inc: Employee|Pfizer Inc: Stocks/Bonds **Heather Rubino, PhD, MS**, Pfizer: Stocks/Bonds **Victoria Hendrick, BSc**, Pfizer: Employee|Pfizer: Stocks/Bonds **Holly Soares, PhD**, Pfizer: Stocks/Bonds **Jennifer Hammond, PhD**, Pfizer: Employee|Pfizer: Stocks/Bonds

